# Arthroscopic Excision of Scapular Exostoses: A Technical Note

**DOI:** 10.3390/jcm14072464

**Published:** 2025-04-04

**Authors:** Felix Hochberger, Kilian List

**Affiliations:** Department of Orthopaedic Surgery, Julius-Maximilians University Wuerzburg, Koenig-Ludwig-Haus, Brettreichstrasse 11, 97074 Wuerzburg, Germany; felix.hochberger@klh.de

**Keywords:** arthroscopic surgery, scapular exostoses, minimally invasive technique, shoulder surgery, bone excision

## Abstract

**Background**: Cartilaginous exostoses of the scapula are rare and can cause symptoms such as pain and mechanical crepitus due to scapulothoracic bursitis. While open surgical resection remains the standard approach, it is associated with significant tissue disruption and longer rehabilitation. This technical note presents a minimally invasive arthroscopic technique for excising scapular exostoses. **Methods**: We report the case of a 22-year-old female patient with a symptomatic ventral scapular exostosis. After confirmation of a benign lesion, the exostosis was excised en bloc under continuous arthroscopic guidance. The surgical procedure, including patient positioning, portal placement, instrumentation, and specimen removal, is described in detail. **Results**: The lesion was successfully excised as a single piece for histopathological analysis. The patient experienced no intra- or postoperative complications. Postoperative rehabilitation included early passive motion, and full recovery was achieved within six weeks. At 24-month follow-up, the patient remained pain-free with complete restoration of shoulder function and no evidence of recurrence. **Conclusions**: Arthroscopic excision of scapular exostoses offers a viable alternative to open surgery. The technique minimizes soft tissue trauma, supports faster recovery, and may be safely performed in experienced hands with appropriate preoperative planning and imaging.

## 1. Case Presentation

**Figure 1:**
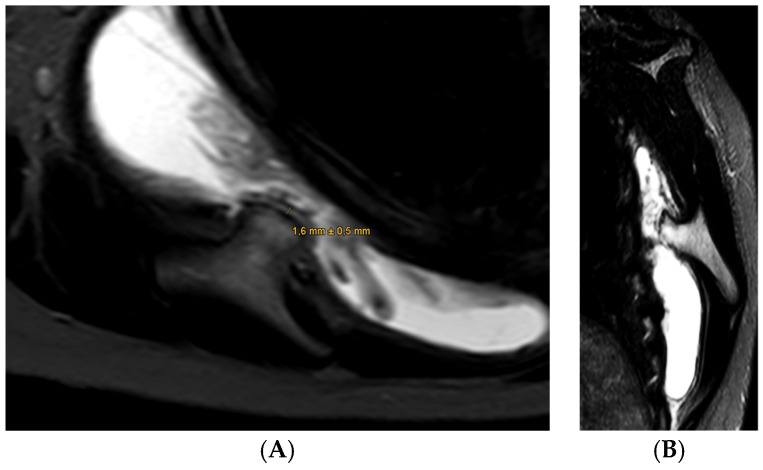
T1 axial (**A**) and T1 sagittal (**B**) views of the scapula showing a cartilaginous exostosis covered by a thin cartilage cap of 1.6 mm on the ventral scapular plane. A concomitant inflammation of the scapulothoracic bursa (presented in white color) is seen on both images.

A 22-year-old female patient presented to our clinic with a chief complaint of a “clicking” sensation in her right shoulder, without any preceding trauma. She reported little pain. Her medical history was unremarkable, with no prior surgeries or injuries. Clinical examination revealed full active and passive range of motion with little pain but a notable scapulothoracic dyskinesis on the right side with reproducible “clicking”. Her shoulder girdle musculature was well-developed, without atrophy. Neurological examination was without pathological findings. Conventional radiography demonstrated age-appropriate findings. MRI subsequently revealed mild irritation of the subacromial bursa with otherwise normal intra-articular structures (Figure 1). In the lower sequences, significant bursitis was noted between the scapula and the thoracic wall, accompanied by a suspicious bony mass (13 mm × 14 mm) covered by a thin cartilage cap (1.6 mm) on the ventral scapular blade. Given the suspicion of a bony lesion, a CT scan was performed, confirming the presence of a broad-based osteochondral exostosis. Based on the radiographic findings, differential diagnoses included benign lesions such as periosteal chondroma or Nora ’s lesion (bizarre parosteal osteochondromatous proliferation), as well as low-grade peripheral chondrosarcoma. The case was discussed in the institutional tumor board conference, which confirmed a benign bony exostosis. The patient’s symptoms indicated the resection of the exostosis. Regarding the absence of any signs of malignancy and the location on the central scapular plane, we decided to deviate from the tumor surgery protocol and to perform a minimal invasive arthroscopic approach to remove the exostosis.

To better illustrate the rationale behind choosing an arthroscopic approach in this case, we provide a comparison of the key clinical parameters associated with open versus arthroscopic excision techniques for scapular exostoses. This overview highlights the potential advantages of the minimally invasive method in terms of operative efficiency, recovery, and patient comfort (Table 1).

## 2. Introduction

Cartilaginous exostoses are benign bone tumors that typically arise from the metaphysis of long bones. They are most commonly located at the proximal humerus or distal femur [4]. With an incidence of 1 in 50,000 [5], solitary cartilaginous exostoses are the most common benign tumors of the musculoskeletal system, accounting for approximately 40% of all cases and about 20% of all bone tumors in humans [5]. Females are less frequently affected by cartilaginous exostoses compared to males [6]. In addition to the primary locations previously mentioned, cartilaginous exostoses frequently affect the pelvis, ribs, scapula, and phalanges [6]. Cartilaginous exostoses are typically covered by a thin cartilage cap, typically growing in a stalk-like, mushroom-shaped manner, and tend to move away from the nearest joint as they expand [7]. They can occur as solitary or as multiple growths at various locations in the skeleton. Their frequent occurrence suggests the diagnosis of hereditary multiple exostosis (HME) [2]. HME is thought to result from a failure in normal endochondral ossification. This process is driven by an imbalance in chondrocyte proliferation and differentiation, as well as aberrant extracellular matrix signaling. Genetic mutations in the EXT1 and EXT2 genes—both tumor suppressor genes—play a central role in the pathogenesis of hereditary multiple exostoses (HME) [4]. These genes encode glycosyltransferases that are essential for the biosynthesis of heparan sulfate, a key component of the extracellular matrix [8]. Impaired heparan sulfate production disrupts the gradient and signaling of crucial morphogenetic proteins such as fibroblast growth factors (FGFs), bone morphogenetic proteins (BMPs), and Indian hedgehog (IHH), all of which regulate growth plate organization and chondrocyte proliferation [9]. As a consequence, excessive and disorganized cartilage proliferation occurs, which then undergoes endochondral ossification, leading to the formation of exostoses [10]. This mechanism is particularly evident in patients with hereditary multiple exostoses, where multiple lesions are present, but it may also play a role in the development of solitary osteochondromas. Furthermore, studies have demonstrated increased osteoblastic activity and proliferative signaling within the cap of exostoses, contributing to continued lesion growth during skeletal development [11].

The risk of progression or malignant transformation into a secondary peripheral chondrosarcoma is approximately 1% for solitary cartilaginous exostoses and about 5% for multiple cartilaginous exostoses [12]. The majority of cartilaginous exostoses are asymptomatic and are best managed through observation. Symptoms associated with cartilaginous exostoses are primarily related to the size of the lesion and its mass effect. These can include pain caused by fractures, bursa formation—as in the present case—or impingement of tendons or nerves [3]. The association between cartilaginous exostoses of the ventral scapula and scapulothoracic impingement has been previously described [4].

While open excision remains the standard surgical treatment for symptomatic scapular exostoses, it typically requires extensive soft tissue dissection, including detachment of the trapezius and rhomboid muscles, which can result in prolonged recovery times, muscle weakness, and postoperative complications such as scapular dyskinesis [1]. Recent case reports and small series have suggested that arthroscopic techniques can minimize soft tissue trauma and facilitate faster postoperative rehabilitation [13,14,15,16]. However, there is a lack of standardized surgical protocols or detailed technical descriptions that can guide orthopedic surgeons in safely performing these procedures. Additionally, most of the existing literature either lacks comprehensive reporting on intraoperative steps or is limited to highly selected cases. This technical note seeks to address this gap by providing a step-by-step description of an arthroscopic technique for ventral scapular exostosis excision, emphasizing safety, reproducibility, and oncological principles such as specimen harvest for histopathology.

## 3. Surgical Technique

**Figure 2:**
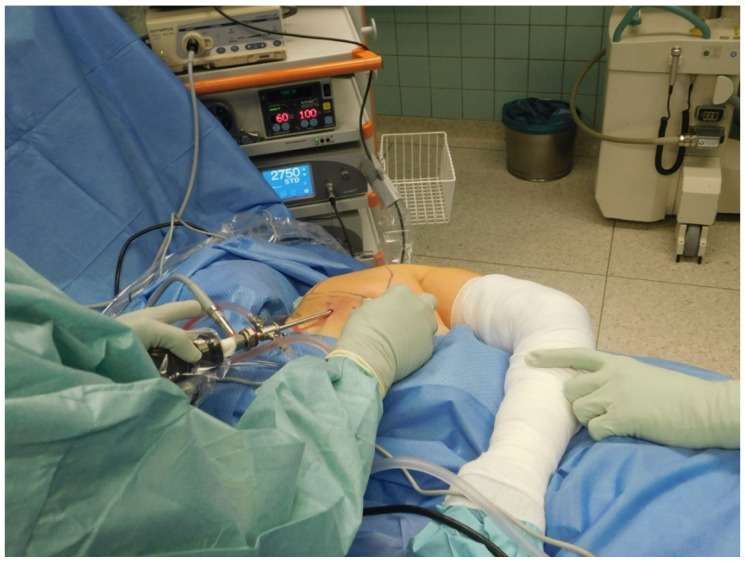
The figure shows the left shoulder of a female patient in the standard prone position with the arm in an adducted and internally rotated position. The anatomical landmarks were drawn, and the arthroscopic approach via the posterior visualization portal was chosen.

Patient Positioning and Setup: The patient is positioned prone on a standard operating table with the affected arm placed in internal rotation, resting behind the back (Figure 2). This posture elevates the medial scapular border, improving access to the scapulothoracic space. General anesthesia with endotracheal intubation is performed. A water pressure pump is used to maintain continuous irrigation at 60 mmHg with 100% flow settings. A 5 mm 30° arthroscope (Arthrex, Naples, FL, USA) is introduced, and standard arthroscopic instruments, including a 4 mm shaver and a vapor electrode (radiofrequency ablation), are prepared. Preoperative antibiotic prophylaxis is administered using amoxicillin/clavulanic acid.

Portal placement: Three portals are utilized to access the ventral scapular surface:

- Posterior portal (visualization): Located 2 cm medial and inferior to the posterolateral acromial border.
- Lateral portal: Placed over the lateral scapular border for instrumentation or triangulation.
- Anterior portal: Established under direct visualization when additional access is needed for retraction or instrument guidance.

**Figure 3:**
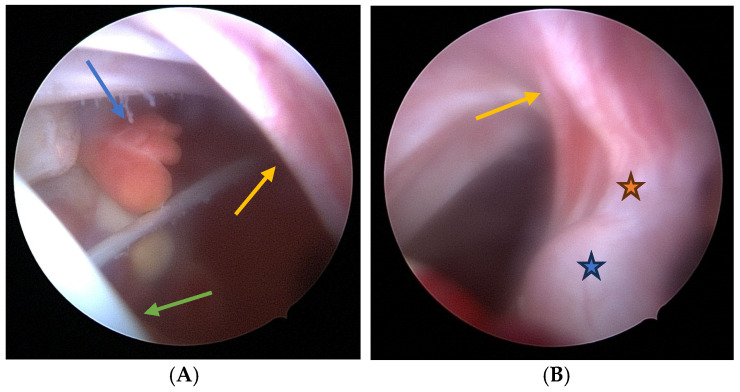
Arthroscopic view showing the inflamed scapulothoracic bursa (**A**,**B**) caused by a cartilaginous exostosis located on the ventral surface of the scapular blade. The scapular blade with the parietal bursal layer is marked by the yellow arrow, the thoracic cavity by the green arrow, and the inflamed bursal tissue by the blue arrow. The neck of the exostosis is indicated by the orange star, and the main body of the exostosis is indicated by the blue star.

**Figure 4:**
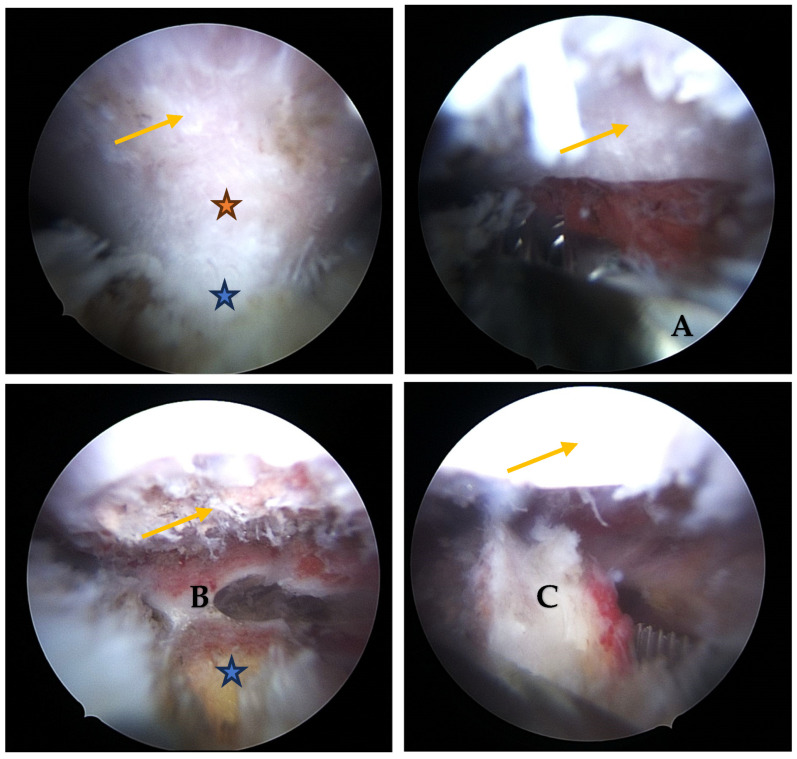
Arthroscopic resection of a cartilaginous exostosis covered with synovial membrane, located on the ventral aspect of the scapular blade. All images are oriented with the scapular surface positioned superiorly and the exostosis body inferiorly. Yellow arrow: scapular blade; orange star: neck of the exostosis; blue star: main body of the exostosis. (**A**) Grasp used to resect the exostosis at the neck; (**B**) final resection plane; (**C**) grasping instrument holding the fully resected exostosis.

**Figure 5:**
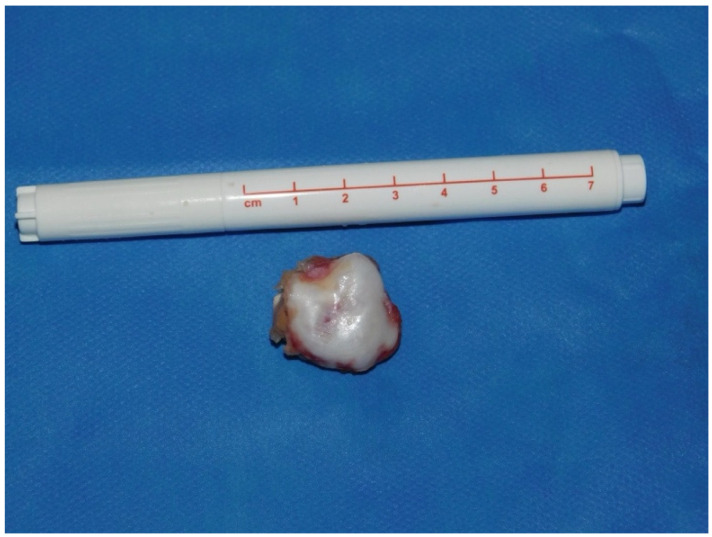
Figure showing an arthroscopically removed cartilaginous exostosis with its thin cover of cartilage.

Exostosis excision:

1. Visualization: The arthroscope is introduced through the posterior portal. Initial visualization targets the inflamed scapulothoracic bursa to gain orientation (Figure 3A,B). In the present case, the exostosis was partially protruding through the subscapularis muscle and thus visible upon entry. A blunt dissection plane is established between the scapula and the subscapularis muscle to expose the exostosis base.
2. Instrumentation: A second posterior portal is established for working access. Through this portal, arthroscopic burrs, graspers, and the electrosurgical device are inserted. In certain cases, lateral or anterior accessory portals may enhance triangulation or access to deeper portions of the scapula.
3. Resection: Using a high-speed burr under continuous irrigation, the lesion is carefully resected flush to the scapular surface. To ensure oncologic safety, the exostosis is excised at its base as a single piece and removed using an arthroscopic grasper for histopathological examination (Figure 4). Constant visualization is maintained to avoid iatrogenic injury to adjacent structures.
4. Histopathological Confirmation: The excised specimen (Figure 5) is sent for formal pathological analysis. In our case, the diagnosis of benign osteochondroma was confirmed without atypical or malignant features.
5. Hemostasis and Debridement: Electrocautery (vapor electrode) is used to control minor bleeding. Loose bone fragments and inflamed bursal tissue are debrided thoroughly using a 4 mm shaver and suction.
6. Closure: All portals are closed with absorbable sutures. Sterile dressings are applied, and the patient is placed in a sling for comfort.

## 4. Postoperative Rehabilitation and Outcomes

Postoperative care includes pain management, immobilization in a sling for the first week, and initiation of passive range of motion exercises within the first two weeks. Patients are advised to avoid strenuous activities for six weeks post-surgery.

Early results indicate a reduction in postoperative pain and fast recovery times. Patients report high satisfaction with improved shoulder function and minimal scarring. The patient presented here had a VAS of 0, a Constant–Murley score of 100, and a QuickDASH of 95 points at 24 months follow-up. There was no evidence of local recurrence.

In our index case, the patient demonstrated full recovery with complete pain relief and restoration of shoulder function at 24 months follow-up. While this technical note is centered on a single clinical case, the described technique has been successfully applied to two patients at our institution. Both patients reported resolution of mechanical symptoms and returned to full daily activity within six weeks postoperatively. No intra- or postoperative complications were observed. Previously, patients with similar diagnoses were primarily treated through open surgery. These findings are in line with published case reports and small case series, which describe excellent functional outcomes following arthroscopic resection of ventral scapular exostoses, especially in young, active patients [1,2,16]. Fageir et al. [1] and Alshayhan et al. [2] reported similar improvements in pain, function, and satisfaction following both open and arthroscopic approaches, with a tendency toward quicker recovery in the arthroscopy group. While higher-level evidence is currently limited, the consistency across reports suggests that this minimally invasive technique may be a viable alternative to open surgery in appropriately selected cases. These results are further discussed in comparison with the literature in the following section.

## 5. Risks and Technical Challenges

Although the arthroscopic approach offers several advantages over traditional open excision, it is not without risk. One of the main challenges is the limited visualization due to the complex topography of the scapulothoracic space. Incomplete resection may occur if the base of the exostosis is not adequately exposed, potentially leading to recurrence of symptoms [17]. Iatrogenic injury to the dorsal scapular nerve or accessory nerve is a known risk, especially during portal placement or aggressive dissection near the medial scapular border [18]. Surgeons must be familiar with the regional anatomy and utilize intraoperative landmarks to minimize this risk. Fluid extravasation into the surrounding soft tissues may also cause postoperative swelling or discomfort, particularly if irrigation pressure is not carefully controlled. Moreover, due to the rarity of the procedure, technical proficiency and familiarity with arthroscopic scapulothoracic anatomy are critical. The learning curve should not be underestimated. To mitigate these complications, the use of detailed preoperative imaging (CT or MRI), careful portal planning, and continuous visualization during burring are essential. In cases of unclear anatomy or suspicious lesion characteristics, an open approach should be favored to ensure complete and safe resection.

## 6. Discussion

The current technique describes our standard arthroscopic procedure for the resection of a cartilaginous exostosis located on the ventral plane of the scapula. Surgical excision of a cartilaginous exostosis is recommended when pain is present [15]. The removal of scapular exostoses can be achieved through either open surgery or arthroscopy. The literature describes various techniques for the excision of cartilaginous exostoses, including both open and arthroscopic methods [17]. To access the anterior plan of the scapula in an open fashion, the trapezius and rhomboideus muscle must be detached, causing substantial soft tissue damage and risking iatrogenic scapula dyskinesis. Arthroscopic excision is generally associated with superior outcomes, such as faster recovery and a reduced rate of complications compared to open excision [3]. However, the arthroscopic approach is technically demanding due to the paucity of clear anatomical landmarks and poses risks such as potential injury to the accessory and dorsal scapular nerves [3]. Arthroscopic excision is reserved for patients where diagnostic workup clearly indicates cartilaginous exostoses and is most beneficial if they are located at the anterior scapula plane. For those located at the margin of the scapula, open access might be as effective. It is contraindicated in cases where there is any suspicion of malignancy to prevent the potential spread of the tumor throughout the bursa or the approach [18]. Although the arthroscopic approach is generally safe, surgeons must remain aware of potential complications such as neurovascular injury, insufficient visualization, or fluid extravasation. In cases where anatomic orientation is unclear or access to the lesion is inadequate, conversion to an open procedure should be considered, for example, to allow for direct nerve exploration or to ensure complete resection. In our assessment, the risk of iatrogenic pneumothorax is negligible when portals are placed correctly. If needed, the use of a double-lumen endotracheal tube may provide additional safety during the procedure.

Fageir et al. showed that the surgical excision of cartilaginous exostoses on the ventral plane of the scapula can yield favorable outcomes, particularly in terms of pain relief and functional restoration. The majority of patients reported significant clinical improvement following the procedure [1]. In 2021, Al-Sahajan et al. [2] reported on the management of a 24-year-old male patient who exhibited both anterior and posterior symptomatic exostoses on the scapular wall. Their study detailed the concurrent removal of these exostoses using both open and arthroscopic surgical techniques within a single operative session, providing an opportunity for direct comparison of the outcomes associated with each method [2]. Both surgical approaches resulted in significant pain relief and improvem^ent in shoulder function. However, the arthroscopic resection offered benefits in terms of postoperative recovery time and the minimally invasive nature of the procedure. While open excision was also effective, it was associated with a longer recovery period and a higher risk of postoperative complications [2]. The patients we treat in the described fashion demonstrated excellent restoration of shoulder function with complete resolution of pre-existing crepitations, total pain relief, and high subjective well-being after 24 months, with no evidence of postoperative complications.

## 7. Limitations

If the benign nature of the lesion is morphologically beyond doubt, the arthroscopic approach offers clear advantages. Nevertheless, it also comes with limitations. The technique requires advanced arthroscopic skills due to the confined scapulothoracic space and limited visualization. Incomplete resection is a risk, especially in cases with broad or irregular lesion bases. Furthermore, the learning curve is significant, and the procedure may not be suitable for all surgeons or institutions. Patient selection is also critical. Lesions with complex anatomy or proximity to neurovascular structures may necessitate open resection for safety and completeness.

We acknowledge that the present report is limited by its single-case design. Although supplemented by our internal experience and supported by similar cases in the literature, the generalizability of this technique cannot be definitively assessed based on this level of evidence. Future prospective studies with larger cohorts are needed to further validate the clinical outcomes and reproducibility of the described approach.

## 8. Conclusions

Arthroscopic excision of scapular exostoses is a viable and effective alternative to open surgery, offering reduced patient morbidity and enhanced recovery. This technique, while demanding a high level of surgical skill, provides promising results in the management of symptomatic scapular exostoses. However, the exclusion of any suspect of malignancy, ideally in an interdisciplinary tumor conference, is crucial when deviating from standards of tumor surgery.

## Figures and Tables

**Table 1:** Comparison of open vs. arthroscopic resection of scapular exostoses.

Parameter	Open Technique	Arthroscopic Technique
Operative Time	Typically longer due to extensive exposure and muscle detachment [1]	Generally shorter with targeted access and minimal dissection [2]
Tissue Disruption	Significant; involves detachment of major muscles, leading to potential weakness and longer recovery [1]	Minimal; preserves muscle integrity, reducing the risk of postoperative dysfunction [2]
Complication Rate	Higher risk of infection, nerve injury, and scapular dyskinesis [1]	Lower complication rates with reduced risk of neurovascular damage and soft tissue trauma [3]
Recovery Time	Extended rehabilitation required; delayed return to daily activities [1]	Accelerated rehabilitation; earlier return to function reported in small series and case reports [2,3]
Postoperative Pain	Increased due to larger incisions and muscle dissection [1]	Reduced postoperative pain attributed to minimal invasiveness and smaller incisions [2]
Tissue Containment	Follows principles of tumor surgery with en bloc resection and controlled tissue handling	Potential dissemination of tumor tissue into the subscapular bursa due to fragmenting or shaving techniques
